# Reconstructing the early global dynamics of under-ascertained COVID-19 cases and infections

**DOI:** 10.1186/s12916-020-01790-9

**Published:** 2020-10-22

**Authors:** Timothy W. Russell, Nick Golding, Joel Hellewell, Sam Abbott, Lawrence Wright, Carl A. B. Pearson, Kevin van Zandvoort, Christopher I. Jarvis, Hamish Gibbs, Yang Liu, Rosalind M. Eggo, W. John Edmunds, Adam J. Kucharski, CMMID COVID-19 working group, Arminder K. Deol, C. Julian Villabona-Arenas, Thibaut Jombart, Kathleen O’Reilly, James D. Munday, Sophie R. Meakin, Rachel Lowe, Amy Gimma, Akira Endo, Emily S. Nightingale, Graham Medley, Anna M. Foss, Gwenan M. Knight, Kiesha Prem, Stéphane Hué, Charlie Diamond, James W. Rudge, Katherine E. Atkins, Megan Auzenbergs, Stefan Flasche, Rein M. G. J. Houben, Billy J. Quilty, Petra Klepac, Matthew Quaife, Sebastian Funk, Quentin J. Leclerc, Jon C. Emery, Mark Jit, David Simons, Nikos I. Bosse, Simon R. Procter, Fiona Yueqian Sun, Samuel Clifford, Katharine Sherratt, Alicia Rosello, Nicholas G. Davies, Oliver Brady, Damien C. Tully, Georgia R. Gore-Langton

**Affiliations:** 1 Centre for Mathematical Modelling of Infectious Diseases, London School of Hygiene & Tropical Medicine, London, UK; 2 Telethon Kids Institute and Curtin University, Perth, Western Australia Australia; 3 Defence Science and Technology Laboratory/Sopra Steria, Fareham, UK

**Keywords:** Case ascertainment, COVID-19, SARS-CoV-2, Surveillance, Under-reporting, Situational awareness, Outbreak analysis

## Abstract

**Background:**

Asymptomatic or subclinical SARS-CoV-2 infections are often unreported, which means that confirmed case counts may not accurately reflect underlying epidemic dynamics. Understanding the level of ascertainment (the ratio of confirmed symptomatic cases to the true number of symptomatic individuals) and undetected epidemic progression is crucial to informing COVID-19 response planning, including the introduction and relaxation of control measures. Estimating case ascertainment over time allows for accurate estimates of specific outcomes such as seroprevalence, which is essential for planning control measures.

**Methods:**

Using reported data on COVID-19 cases and fatalities globally, we estimated the proportion of symptomatic cases (i.e. any person with any of fever ≥ 37.5 °C, cough, shortness of breath, sudden onset of anosmia, ageusia or dysgeusia illness) that were reported in 210 countries and territories, given those countries had experienced more than ten deaths. We used published estimates of the baseline case fatality ratio (CFR), which was adjusted for delays and under-ascertainment, then calculated the ratio of this baseline CFR to an estimated local delay-adjusted CFR to estimate the level of under-ascertainment in a particular location. We then fit a Bayesian Gaussian process model to estimate the temporal pattern of under-ascertainment.

**Results:**

Based on reported cases and deaths, we estimated that, during March 2020, the median percentage of symptomatic cases detected across the 84 countries which experienced more than ten deaths ranged from 2.4% (Bangladesh) to 100% (Chile). Across the ten countries with the highest number of total confirmed cases as of 6 July 2020, we estimated that the peak number of symptomatic cases ranged from 1.4 times (Chile) to 18 times (France) larger than reported. Comparing our model with national and regional seroprevalence data where available, we find that our estimates are consistent with observed values. Finally, we estimated seroprevalence for each country. As of 7 June, our seroprevalence estimates range from 0% (many countries) to 13% (95% CrI 5.6–24%) (Belgium).

**Conclusions:**

We found substantial under-ascertainment of symptomatic cases, particularly at the peak of the first wave of the SARS-CoV-2 pandemic, in many countries. Reported case counts will therefore likely underestimate the rate of outbreak growth initially and underestimate the decline in the later stages of an epidemic. Although there was considerable under-reporting in many locations, our estimates were consistent with emerging serological data, suggesting that the proportion of each country’s population infected with SARS-CoV-2 worldwide is generally low.

## Background

The pandemic of the novel coronavirus SARS-CoV-2 has caused 25.3 million confirmed cases and 846,841 deaths as of 31 August 2020 [1]. As a precautionary measure, or in response to locally detected outbreaks, countries have introduced control measures with varying degrees of stringency [1], including isolation and quarantine, school and workplace closures, bans on social gatherings, physical distancing and face coverings, and stay-at-home orders [2, 3]. Several features of SARS-CoV-2 make accurate detection during an ongoing epidemic challenging [4–6], including high transmissibility [3, 7, 8], an incubation period with a long-tailed distribution [9], pre-symptomatic transmission [10], and the existence of asymptomatic infections, which may also contribute to transmission [11]. These attributes mean that infections can go undetected [12] and that countries may only detect and report a fraction of their infections [3, 13].

Understanding the extent of unreported infections in a given country is crucial for situational awareness. If the true size of the epidemic can be estimated, this enables a more reliable assessment of how and when non-pharmaceutical interventions (NPIs) should be both introduced, as infections rise, or relaxed as infections fall [3]. Estimates of infection prevalence are also important for obtaining accurate measures of transmission: if the proportion of infections reported declines as the epidemic rises, the number of confirmed cases will grow slower than the actual underlying epidemic. Likewise, if detection rises as the epidemic declines, it may appear that transmission is not declining as fast as it is in reality. Underdetection of cases also makes it challenging to estimate at what stage of the epidemic a particular country is [14]: viewed in isolation, case incidence data could reflect a very large undetected epidemic, or a smaller, better reported epidemic.

To estimate how the levels of under-ascertainment vary over time, we present a modelling framework that combines data on reported cases and deaths, and published severity estimates. We apply our methods to countries that have reported more than ten deaths to date, then use these under-ascertainment estimates to reconstruct global epidemics in all countries where case and death time series data are available. We also compare the model estimates for cumulative incidence against existing seroprevalence results. Finally, we present the adjusted case curves for the ten countries with the highest confirmed and adjusted case numbers, as well as global prevalence estimates for SARS-CoV-2.

## Methods

As SARS-CoV-2 infections that generate mild symptoms are more likely to be missed than severe cases, the ratio of cases to deaths, adjusting for delays from report to fatal outcome, can provide information on the possible extent of undetected symptomatic infections. Using a Bayesian Gaussian process model, we estimate changes in under-ascertainment over time, as described below.

### Adjusting for delay from confirmation to death

In real time, simply dividing deaths to date by cases to date leads to a biased estimate of the case fatality ratio (CFR), because this naive calculation does not account for delays from confirmation of a case to death, and under-ascertainment of cases [5, 6] and in some circumstances, under-ascertainment of deaths too. Using the distribution of the delay from hospitalisation to death for cases that are fatal, we can estimate how many cases so far are expected to have known outcomes (i.e. death or recovery), and hence adjust the naive estimates of CFR to account for these delays and produce a delay-adjusted CFR (dCFR). Separately published dCFR estimates for a given country can be used to estimate the number of symptomatic cases that would be expected for a given dCFR trajectory. Available estimates for the CFR that adjust for under-reporting typically range from 1 to 1.7% [7–10]. Large studies in China and South Korea estimate the CFR at 1.38% (95% CrI 1.23–1.53%) [9] and 1.7% (95% CrI 1.1–2.5%) [7] respectively.

### Inferring level of under-ascertainment

Assuming a baseline CFR of 1.4% (95% CrI 1.2–1.5%), the ratio of this baseline CFR to our estimate of the dCFR for a given country can be used to derive a crude estimate of the proportion of symptomatic cases that go unreported for this country. For each country, we calculate the dCFR on each day and use the ratio of the baseline CFR to the dCFR estimate to produce daily estimates of the proportion of unreported cases. We then use a Gaussian process (GP) model to fit a time-dependent under-ascertainment rate for each country. A more detailed description of the methods, including the mathematical details of the Gaussian process and the different sources of uncertainty present in the model, can be found in the Supplementary Material.

With the aim of developing a parsimonious and easily transferable analysis framework, we assume the same baseline CFR for all countries in the main results. Given that CFR varies substantially with age [5], this induces a certain amount of error in our estimates, especially for countries with age distributions significantly different to China, where the data used to derive the baseline CFR estimates originated [5]. Therefore, we include a version of all the main results where we compute an indirectly adjusted baseline CFR, using the underlying age distribution of each country using the wpp2019 R package [15] and the age-stratified CFR estimates from [5] in the supplementary material (Additional file 1: Figure S5, S6 and S7), where we also include a verbose limitations section discussing at length the potential errors induced under such assumptions.

### Relationship between under-ascertainment and testing

We attempt to characterise the relationship between widespread RT-PCR testing and case ascertainment using our temporal under-ascertainment estimates and testing data for many countries from OurWorldInData [16]. We do so by performing a correlation test between the two for all countries we had both data for. The resulting bivariate scatterplot is included in the supplementary material (Additional file 1: Figure S3).

### Comparison against seroprevalence estimates

**Fig. 1:**
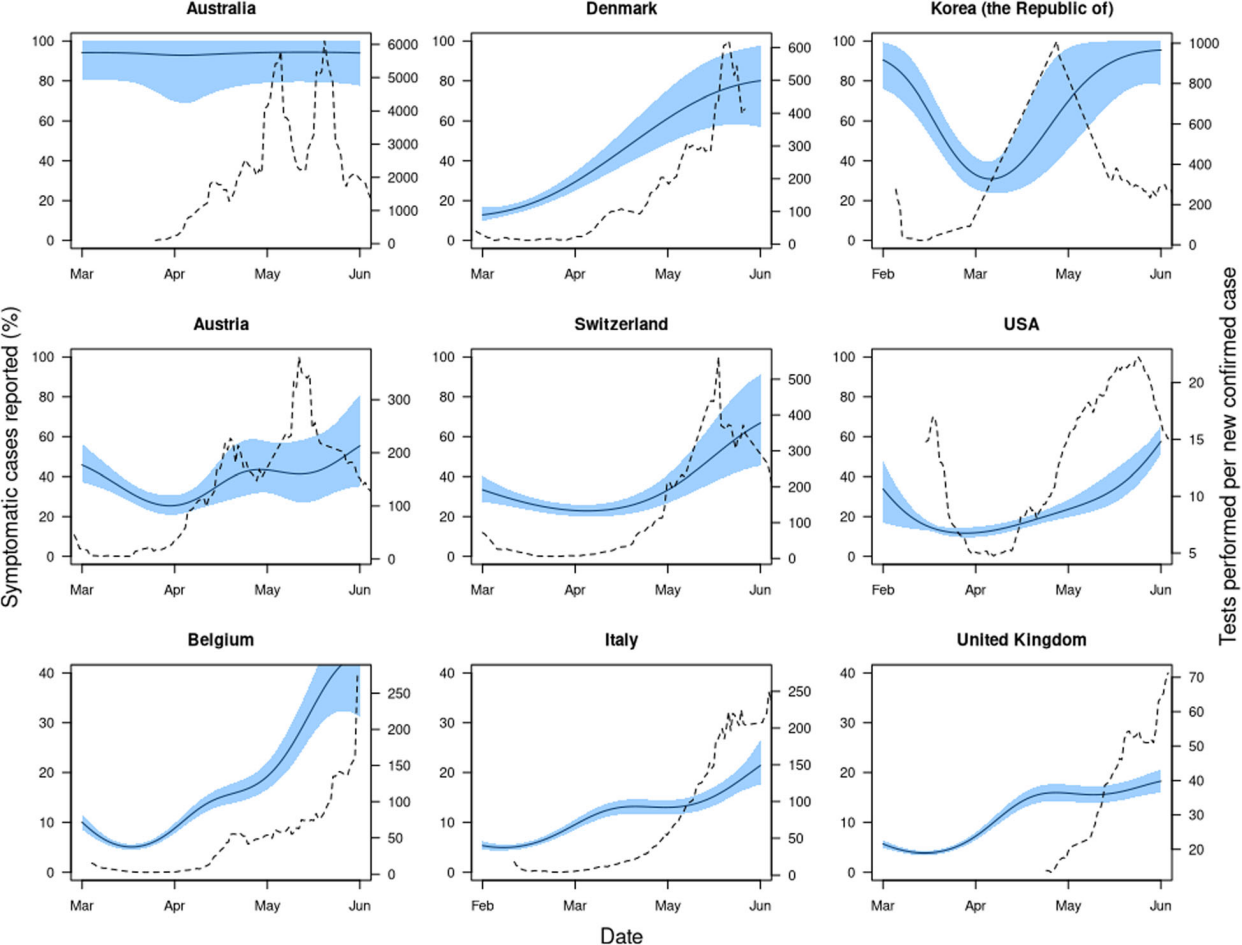
Illustrative examples of temporal variation in under-ascertainment and testing effort. Nine countries under-ascertainment and testing effort dynamics, where the under-ascertainment dynamics display a typical U-trend. The solid black line is the estimated median proportion of symptomatic cases ascertained over time and the shaded blue region is the 95% credible interval of these ascertainment estimates. Dashed line shows the reported testing effort, which we defined as a 7-day moving average of the number of new tests per new case reported each day. The illustrative examples chosen in Fig. 1 were constrained by the availability of testing data over a time period comparable to our under-ascertainment estimates. However, all countries under-ascertainment estimates, with or without testing data, are presented in Additional file 1: Figure S1

We attempted to reconstruct the infection curves by first adjusting the reported case data for under-ascertainment (Fig. 1). We then adjust further these estimated symptomatic case curves so that they represent all infections. We do so using the assumption that 50% of infections are asymptomatic (with an assumed wide range of 10-70% feeding into our estimates 95% credible interval) and mean-lagging the time point to adjust for the delay between onset of symptoms and confirmation [18]. We assume that serological tests are broadly similar between locations, in order for a tractable and relatively simple comparison. We include both our estimates, with their 95% credible intervals, and the confidence intervals of all serological estimates in our comparison (Fig. 3).

### Data and code availability

The data we use is publicly available online from the European Centre for Disease Control (ECDC) [19]. The code for the dCFR and under-reporting estimation model can be found here: https://github.com/thimotei/CFR_calculation. The code to read in the under-ascertainment data and to reproduce the figures in this analysis can be found here: https://github.com/thimotei/covid_underreporting.

## Results

We estimated substantial variation in the proportion of symptomatic cases detected over time in many of the countries considered (Fig. 1 and Figure S1). For example, during March, the median percentage of symptomatic cases detected across the 84 countries which experienced more than ten deaths ranged from 2.38% (Bangladesh) to 99.6% (Chile). Also during March, the median percentage of symptomatic cases detected across Europe ranged from 4.81% (France) to 85.5% (Cyprus).

Countries might expect to detect an increasing proportion of symptomatic cases if they scale up testing effort in response to the outbreak. To measure this, we compared our estimates for the proportion of cases detected with the number of tests performed per new case each day, which can provide an indication of testing effort with a country [19]. Taking a moving average with a 7-day window, we found that countries that showed high testing effort did not necessarily have high levels of case ascertainment. For example, in a 2-week period in March, the UK performed 80 tests per new case (the mean across Europe during the same period was 27 tests per new case). However, we estimate that also in the UK only between 3 and 10% of symptomatic cases were being detected (Fig. 1). Overall, we found a weak positive correlation between testing effort and case ascertainment (Kendall’s correlation coefficient of 0.16). This suggests that increased testing effort can help to improve case ascertainment, but on its own is not enough to guarantee low levels of under-ascertainment.

Using our temporal under-ascertainment trends, we estimate that during March, April, and May the percentage of symptomatic cases detected in European countries and averaged over time ranged from 4.8 to 86% (France–Cyprus), 5.8 to 100% (France–Belarus) and 11 to 86% (Hungary–Cyprus) respectively. By comparison, the number of reported tests performed per new confirmed case, averaged over the month in question, ranged between 2.7 and 76 in March (Belgium–Portugal), 2.7 to 832 in April (Belgium–Slovakia) and 12 to 1334 (Ukraine–Lithuania) in May.

**Fig. 2:**
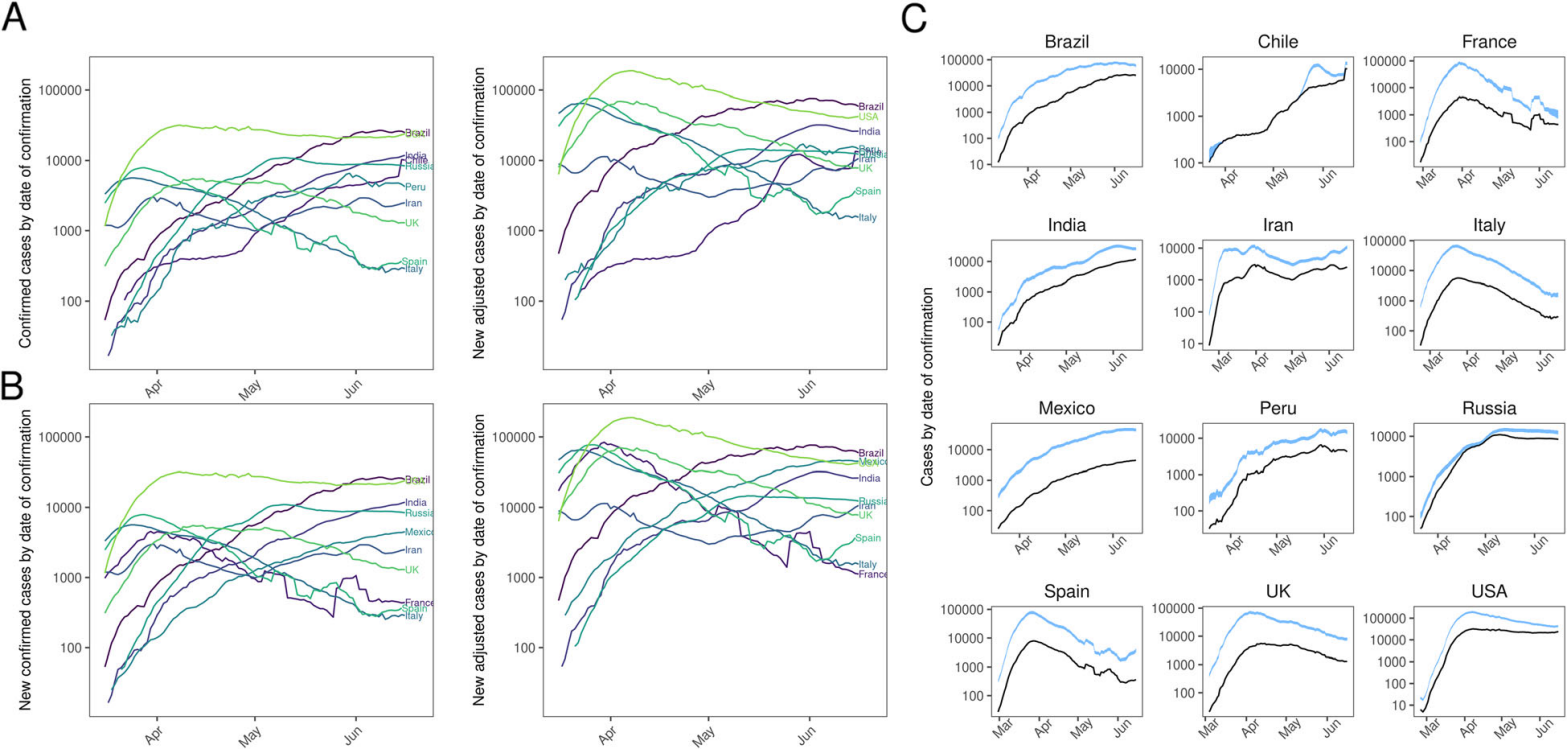
Confirmed case curves adjusted for temporal under-ascertainment. **a** Confirmed cases (left) and adjusted cases (right) for the ten countries with the highest number of confirmed cases. **b** Confirmed cases (left) and adjusted cases (right) for the ten countries with the highest number of confirmed cases after adjusting for under-ascertainment. There are two countries which change between **a** and **b**: France and Mexico are replaced by Chile and Peru respectively. **c** The same curves plotted in **a**, but with a plot per country. Blue-shaded region corresponds to the 95% CrI of the adjusted curves. **a** and **b** highlight between country variation whereas **c** highlights within country variation

Adjusting confirmed case data for under-ascertainment to obtain estimated symptomatic case curves, we found a much larger and more peaked epidemic in the ten countries with the highest total number of confirmed cases and the ten with the highest number of adjusted cases as of 6 July 2020 (Fig. 2, with estimates for other countries shown in Additional file 1: Figure S2). Typically, the estimated peak of symptomatic cases in these countries ranged from 1.4 times (Chile) to 17.8 times larger (France) than the peak in the reported case data (Table 1). Moreover, in the five countries of these ten that had a clear initial peak before the end of May 2020, we estimated that the post-peak decline in the number of infections was steeper than that implied by the confirmed case curves (Fig. 2b).

**Table 1:** Comparison between the confirmed and adjusted case numbers at their respective peaks for ten countries with the highest number of total confirmed cases and ten countries with the highest number of symptomatic cases after adjusting for under-ascertainment. Eight countries are in both lists, so the total is twelve distinct countries. We find that the peak of the case curves shifts when they are adjusted for under-ascertainment. Clearly, Mexico and Brazil have not necessarily peaked yet, given that they are not as far along their epidemic as the other countries. Therefore, for these countries, we simply report the date and number of the highest number of cases to date

	Date		Value at peak	
Location	Peak of confirmed cases	Estimated change in peak date (absolute value)	New confirmed cases at peak	Estimated total cases (95% CrI)
Brazil	6 June 2020	0 days	54,771	122,512 (110,660–137,374)
Chile	18 June 2020	3 days	36,179	52,042 (47,828–56,338)
France	1 April 2020	0 days	7578	134,594 (120,450–151,352)
India	21 June 2020	18 days	15,413	48,513 (43,433–54,939)
Iran	5 April 2020	0 days	5275	17,931 (16,078–20,201)
Italy	22 March 2020	0 days	6557	75,521 (64,229–91,630)
Mexico	13 June 2020	0 days	5222	55,661 (50,204–62,237)
Peru	4 June 2020	0 days	24,603	24,603 (22,121–27,629)
Russia	12 June 2020	4 days	11,656	15,604 (14,248–17,270)
Spain	27 March 2020	1 day	9181	85,881 (77,697–96,319)
UK	12 April 2020	0 days	8719	100,870 (91,054–112,639)
USA	26 April 2020	21 days	48,529	280,631 (226,097–344,472)

**Fig. 3:**
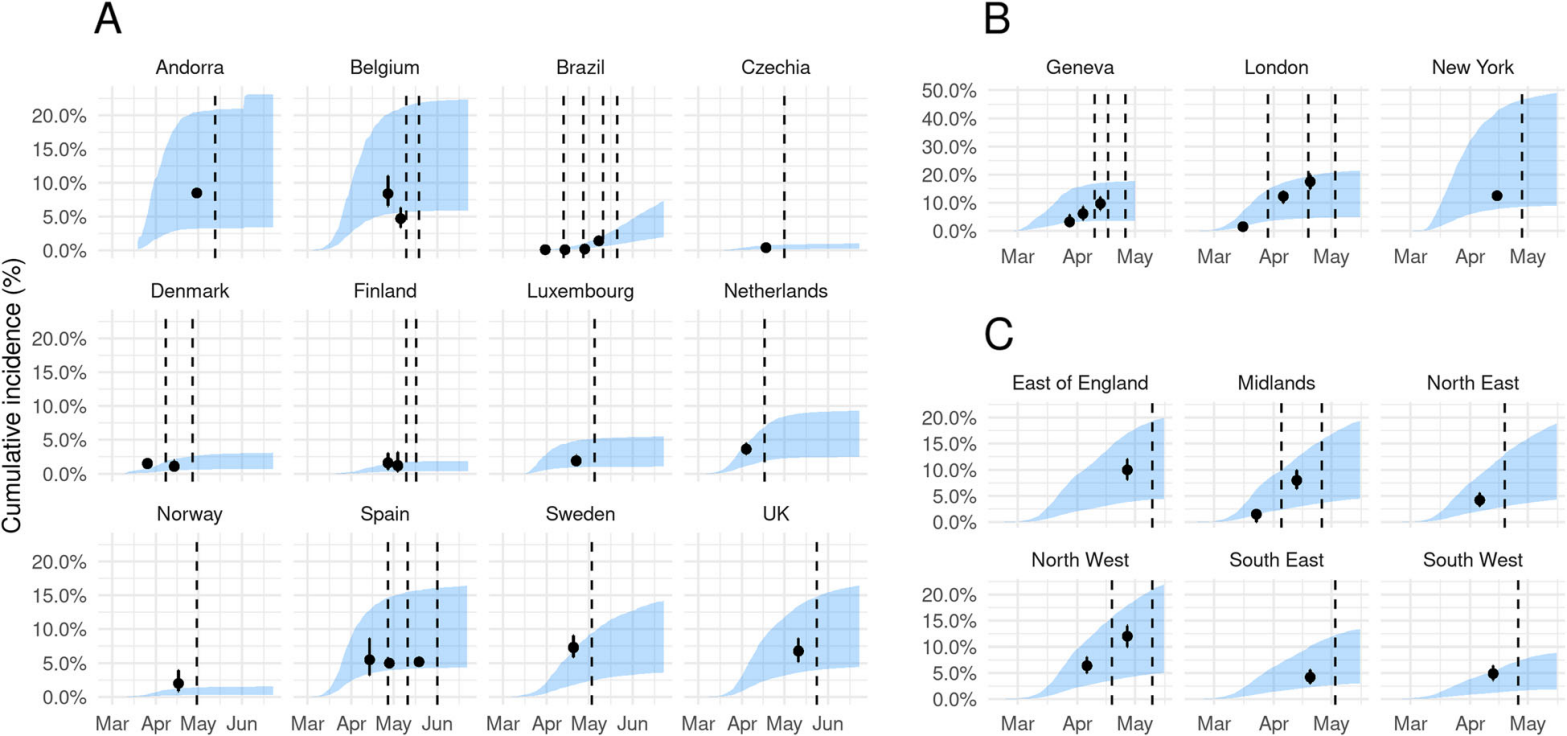
Estimated infection prevalence curves compared with observed seroprevalence data. **a** Country-level comparisons. **b** City-level comparisons for Geneva, London and New York. **c** Regional-level comparisons, using six of the eight regions of England. North West and Yorkshire are aggregated together and London is shown above in **b**: After adjusting the reconstructed new cases per day curves for potential asymptomatic infections and for the delay between onset of symptoms and confirmation, we sum up the cases and divide by the population in each country or region, to estimate the total percentage infected. We are then able to directly compare the model estimates to existing seroprevalence results (black points, with 95% binomial CI above and below). Dashed line shows the end of the serological testing period; therefore, we lag the seroprevalence estimate by the mean delay between infection and seroconversion, which is likely to be around 14 days [17]

We also compared the estimated proportion of individuals infected in our model with seroprevalence studies that measured the prevalence of SARS-CoV-2 antibodies. We represent our cumulative incidence estimates in the same form as the observed serological estimates, as a percentage of the population. This is either the population of the country or the population of some smaller region or sub-region, depending on the serological dataset. We found that all but one of the published seroprevalence estimates fell within the 95% credible interval (CrI) of our estimated cumulative incidence curves over time, with the one exception being Denmark where we underestimated the observed seroprevalence (Fig. 3).

**Fig. 4:**
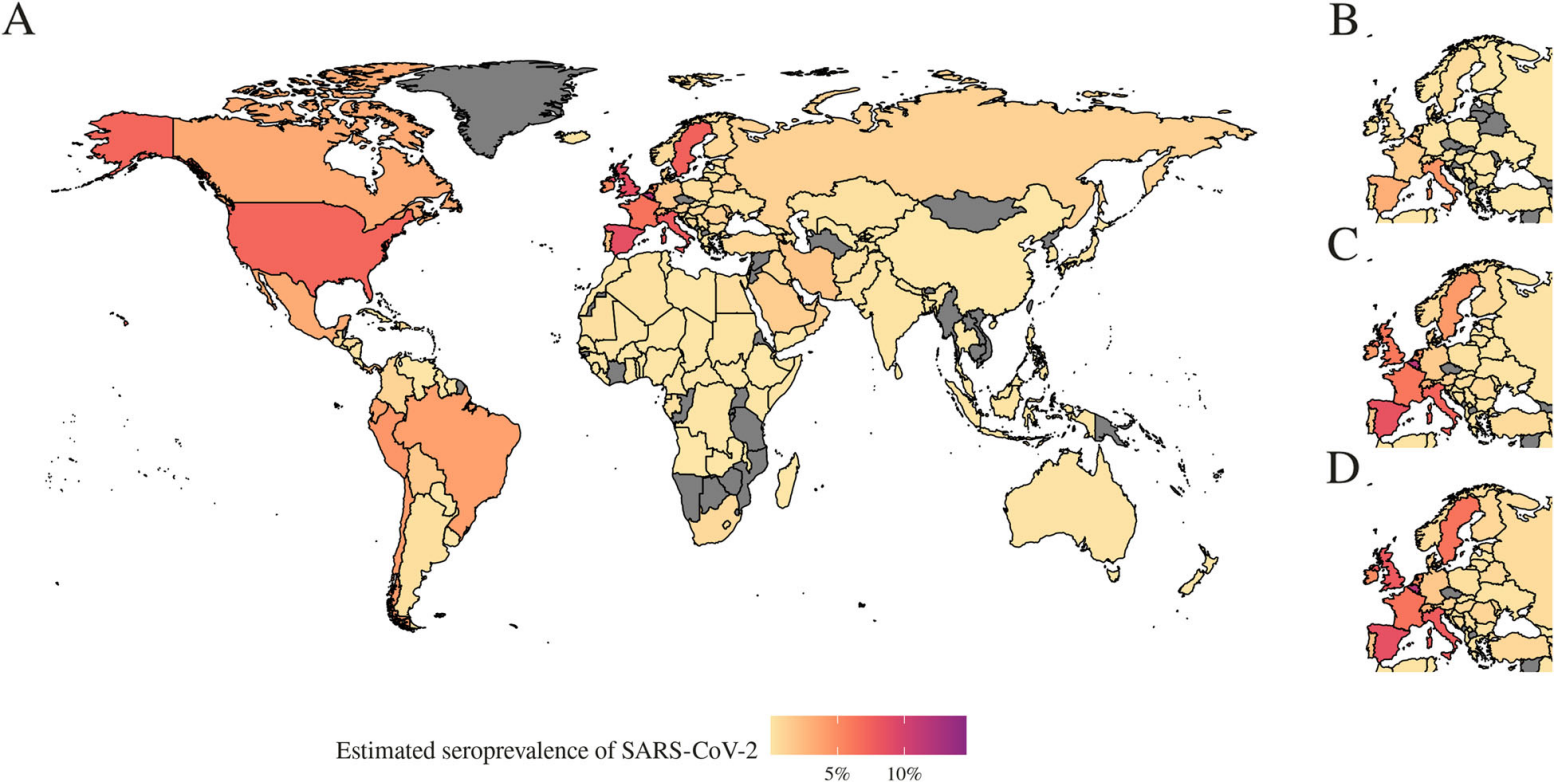
Map of estimated seroprevalence as of the start of June, where we adjusted for under-ascertainment of symptomatic cases and asymptomatic infections. **a** Estimated seroprevalence of SARS-CoV-2 globally as of 7 June 2020, for all countries we have reliable estimates for—greyed out countries represent where we did not have reliable estimates due to insufficient data. **b**–**d** The estimated seroprevalence of SARS-Cov-2 in Europe on **b** 31 March, **c** 30 April and **d** 31 May represent where there was insufficient data to compute reliable estimates

Applying our estimation method to all countries for which case and death time series data are available, we produced a map of seroprevalence estimates as of 16 June (Fig. 4a), suggesting that most infections by this point had been concentrated in Europe and the USA. We estimate that between 0.02 and 15% of populations in Europe have been infected. As of the middle of May, cases were in Latin America and Africa. For both continents combined, we estimate that between 0.00 and 3.48% of the population of these two continents had been infected as of 16 June 2020. We also reconstructed the early progression of the COVID-19 pandemic across Europe (Fig. 4b), finding that the estimated infection prevalence over time was an order of magnitude higher than the confirmed case numbers suggest, with prevalence growing rapidly in late February and early March in several countries.

## Discussion

The epidemiological and clinical characteristics of SARS-CoV-2 mean that a large proportion of infections may go undetected [13, 20]. In the absence of serological data, the ratio between cases and deaths, adjusted for delays from confirmation-to-outcome, can be used to derive estimates of the proportion of symptomatic cases reported. Using this approach, we estimated that case ascertainment dropped substantially in many countries during the peak of their first epidemic wave. Although serological surveys are beginning to emerge [20], many countries do not have such data available, or may only have results from a single cross-sectional survey. The methods and estimates presented here can therefore provide an ongoing picture of the underlying epidemics, including local level dynamics as fine-scale surveillance data become available [21, 22].

Our analysis has some limitations. We assumed the age-adjusted baseline CFR was 1.4% (95% CrI 1.2–1.5%) [4], which is broadly consistent with other published estimates [5, 23, 24], and we assumed a range of 10–70% of infections were asymptomatic [20, 25, 26] with a mean value of 50% [12]. Given the uncertainty in these estimates, we propagated the variance in baseline CFR and range in proportion asymptomatic in the inference process so the final 95% credible interval reported for under-ascertainment reflects underlying uncertainty in the model parameters. We also assumed that deaths from COVID-19 are accurately reported. If local testing capacity is limited, or if testing policy affects attribution of deaths (for example, the evidence for the efficacy of post-mortem swabbing is lacking), deaths can be misattributed to a cause other than COVID-19. In that case, our model may underestimate the true burden of infection. For example, in Peru between 1 April and 1 July 2020, there were 690% excess deaths when compared to confirmed COVID deaths and 3396 reported COVID-19 deaths per 100,000 cases, whereas in the UK there were 199% excess deaths, and 23,642 reported COVID-19 deaths per 100,000. There have also been reports of data reporting issues for several countries [27]. Additionally, if a large proportion of transmission is concentrated within specific age groups, the effective CFR may be higher or lower than the assumed baseline; with better age-stratified temporal data on cases and deaths, it would be possible to explore the effect of this in more detail. However, our estimates were in general consistent with published serological data, where available, providing evidence that our method was robust for these countries at least.

To compare our estimates against seroprevalence studies, and consistent with other simplifying assumptions across countries in this study, we assume that there is little or no variation between the accuracy of the various serological studies included. Including the confidence intervals of each seroprevalence estimate in the comparison allows for some of this variation to be captured quantitatively, but most will be missed. However, as the comparison is crude for a number of reasons, we believe the additional error incurred by such an assumption is minimal. Further, given that our estimates of under-ascertainment in many countries suggest that the numbers of symptomatic infections at the peak of the outbreak were one or two orders of magnitude larger than reported cases, even if deaths are under-reported, our estimates are still likely to be much closer to the true burden than locally reported cases imply.

Our estimates of under-ascertainment over time require a time-series of COVID-19 deaths as an input, a data source that may also exhibit reporting variation. One notable example of this was Spain during June 2020 (Supplementary Appendix: Figure S1). However, as our Gaussian process model quantifies time-varying case ascertainment, it is able to account for positive or negative spikes in reporting [13] (see the Estimating under-ascertainment rates section in the Supplementary Appendix for more details). Finally, our results are limited by the quality of the input data, which is likely to vary in accuracy between countries. However, as we find good agreement between the 95% credible intervals of our estimates and seroprevalence studies, we believe that our model accurately captures some of this variation.

Since the temporal trend in under-ascertainment does not necessarily reflect trends in reported cases or testing effort, evidence synthesis methods such as the one presented here can provide additional insights into whether observed case patterns reflect the underlying epidemic dynamics. In the early stages of outbreaks, this method can provide an indication of whether a large proportion of cases are being detected— and hence whether transmission may be containable with targeted measures such as isolation and contact tracing—or whether transmission is more widespread and a more extensive response is required. Such estimates can also provide insights in the later stages of an outbreak, as they can indicate high levels of detection in countries that have achieved control. For example, in Australia, an adapted version of our model estimated that 80% (95% CrI 55–100%) of cases had likely been ascertained during the outbreak [22]. By adjusting for under-ascertainment, it is also possible to reconstruct the temporal dynamics of SARS-CoV-2 internationally. During February and early March 2020, importations of SARS-CoV-2 into the UK came primarily from Italy, Spain and France [28]. This is consistent with the inferred progression of infection during this period in our model; we estimated that Italy, Spain, France and Belgium all had over 6.5% of the population infected by 31 March 2020 [28].

## Conclusion

Consistent with other studies [3, 20], we estimated that the true numbers of symptomatic cases and infections are appreciably larger than the number of confirmed cases reported (Figs. 1 and 2). We also estimated that the timing of the peak level of symptomatic cases may be considerably earlier or later than the raw confirmed case curve suggests (Table 1). Accurate surveillance of an ongoing outbreak is crucial for estimating key epidemiological values such as the reproduction number, and hence evaluating the impact of control measures [21]. If reported case numbers do not reflect the shape and magnitude of the underlying epidemic, it may bias estimates of transmission potential and effectiveness of interventions. If levels of under-ascertainment are increasing, early interventions may appear to be more effective than they actually are, which could lead to delays in imposing more stringent measures. Likewise, if ascertainment increases in the declining phase of an epidemic, the effectiveness of interventions may be underestimated, potentially leading to measures remaining in place for longer than they would have been had more accurate data been available.

## Supplementary information

**Additional file 1 : Supplementary Appendix 1–4. Figure S1: Figure S1**. Temporal variation in under-reporting for all countries with greater than 10 deaths for more than 50 days. **Figure S2:** Temporal variation in testing effort for all countries there was data for in the Our World In Data database [18]. **Figure S3.** the relationship between case ascertainment and testing effort. We define testing effort as the 7-day moving average of the number of new tests per new case each day. We plot the under-ascertainment estimates along with the testing effort estimates for all countries we have both data for. We then fit, using a loess curve to highlight the positive but weak relationship (, where is Kendall’s rank coefficient). **Figure S4.** Temporal variation in under-ascertainment and testing effort for the nine countries with the maximum total cases that we have reliable testing effort estimates for. This figure differs from Fig. 1 as the results are computed using the indirectly age-adjusted baseline CFR for each country. **Figure S5.** Confirmed case curves adjusted for temporal under-ascertainment adjusted indirectly for age. The results are similar to those in Fig. 2 but have been computed using an indirectly age-adjusted baseline CFR for each country. **Figure S6.** Estimated infection prevalence curves compared with observed seroprevalence data. The results are similar to those in Fig. 3 but have been computed using an indirectly age-adjusted baseline CFR for each country. **Figure S7.** Temporal variation in under-reporting for all countries with greater than 10 deaths for more than 50 days. The results are similar to those in Figure S1 but have been computed using an indirectly age-adjusted baseline CFR for each country. **Table S1.** A summary of the country-level serological studies we used for comparison against our model estimates. **Table S2.** A summary of the city-level or regional-level serological studies we used for comparison against our model estimates. **Table S3.** A summary of the parameters, distributions and output quantities either as inputs or outputs of our under-ascertainment model.

## Data Availability

The data we use is publicly available online from the ECDC [19]. The code for the dCFR and under-reporting estimation model can be found here: https://github.com/thimotei/CFR_calculation. The code to read in the under-ascertainment data and to reproduce the figures in this analysis can be found here: https://github.com/thimotei/covid_underreporting.

## References

[1] Hale T, Webster S, Petherick A, Phillips T, Kira B (2020). Oxford COVID-19 Government Response Tracker. Coronavirus Government Response Tracker.

[2] The effect of large-scale anti-contagion policies on the COVID-19 pandemic | Nature. https://www.nature.com/articles/s41586-020-2404-8. Accessed 1 Sept 2020. 10.1038/s41586-020-2404-8 32512578

[3] Imperial College COVID-19 Response Team, Flaxman S, Mishra S, Gandy A, Unwin HJT, Mellan TA, et al. Estimating the effects of non-pharmaceutical interventions on COVID-19 in Europe. Nature. 2020;584:257–61. 10.1038/s41586-020-2405-7 32512579

[4] Davies NG, Kucharski AJ, Eggo RM, Gimma A, Edmunds WJ, Jombart T, et al. Effects of non-pharmaceutical interventions on COVID-19 cases, deaths, and demand for hospital services in the UK: a modelling study. Lancet Public Health. 2020;5:e375–85. 10.1016/S2468-2667(20)30133-X PMC7266572 32502389

[5] Verity R, Okell LC, Dorigatti I, Winskill P, Whittaker C, Imai N (2020). Estimates of the severity of coronavirus disease 2019: a model-based analysis. Lancet Infect Dis.

[6] Hellewell J, Abbott S, Gimma A, Bosse NI, Jarvis CI, Russell TW (2020). Feasibility of controlling COVID-19 outbreaks by isolation of cases and contacts. Lancet Glob Health.

[7] Abbott S, Hellewell J, Munday J, CMMID nCoV working group, Funk S. The transmissibility of novel Coronavirus in the early stages of the 2019–20 outbreak in Wuhan: Exploring initial point-source exposure sizes and durations using scenario analysis. Wellcome Open Res. 2020 5:17. 10.12688/wellcomeopenres.15718.1 PMC7156988 32322691

[8] Kucharski AJ, Russell TW, Diamond C, Liu Y, Edmunds J, Funk S (2020). Early dynamics of transmission and control of COVID-19: a mathematical modelling study. Lancet Infect Dis.

[9] Lauer SA, Grantz KH, Bi Q, Jones FK, Zheng Q, Meredith HR (2020). The incubation period of coronavirus disease 2019 (COVID-19) from publicly reported confirmed cases: estimation and application. Ann Intern Med.

[10] Tindale L, Coombe M, Stockdale JE, Garlock E, Lau WYV, Saraswat M, et al. Transmission interval estimates suggest pre-symptomatic spread of COVID-19. Epidemiology. 2020. 10.1101/2020.03.03.20029983.

[11] Bai Y, Yao L, Wei T, Tian F, Jin D-Y, Chen L (2020). Presumed asymptomatic carrier transmission of COVID-19. JAMA.

[12] Rivett L, Sridhar S, Sparkes D, Routledge M, Jones NK, Forrest S (2020). Screening of healthcare workers for SARS-CoV-2 highlights the role of asymptomatic carriage in COVID-19 transmission. van der Meer JW, editor. eLife.

[13] Tsang TK, Wu P, Yun Lin YL, Lau E, Leung GM, Cowling BJ. Effect of changing case definitions for COVID-19 on the epidemic curve and transmission parameters in mainland China: a modelling study. The Lancet Public Health. 2020;5:e289–96. 10.1016/S2468-2667(20)30089-X PMC7173814 32330458

[14] Lourenco J, Paton R, Ghafari M, Kraemer M, Thompson C, Simmonds P, et al. Fundamental principles of epidemic spread highlight the immediate need for large-scale serological surveys to assess the stage of the SARS-CoV-2 epidemic. medRxiv. 2020. 10.1101/2020.03.24.20042291.

[15] United Nations Population Division (2020). wpp2019: World Population Prospects 2019. (R package). Available from: https://CRAN.R-project.org/package=wpp2019. Accessed 1 Sept 2020.

[16] Roser M, Ritchie H, Ortiz-Ospina E, Hasell J. Coronavirus pandemic (COVID-19). OurWorldInData.org. 2020. Available from: https://ourworldindata.org/coronavirus. Accessed 1 Sept 2020.

[17] Borremans B, Gamble A, Prager KC, Helman SK, McClain AM, Cox C, Savage V, Lloyd-Smith JO. Quantifying antibody kinetics and RNA detection during early-phase SARS-CoV-2 infection by time since symptom onset. Elife. 2020;9:e60122. 10.7554/eLife.60122 PMC7508557 32894217

[18] He X, Lau EHY, Wu P, Deng X, Wang J, Hao X (2020). Temporal dynamics in viral shedding and transmissibility of COVID-19. Nat Med.

[19] Data on the geographic distribution of COVID-19 cases worldwide. European Centre for Disease Prevention and Control. 2020. Available from: https://www.ecdc.europa.eu/en/publications-data/download-todays-data-geographic-distribution-covid-19-cases-worldwide. Accessed 1 Sept 2020.

[20] Stringhini S, Wisniak A, Piumatti G, Azman AS, Lauer SA, Baysson H, et al. Seroprevalence of anti-SARS-CoV-2 IgG antibodies in Geneva, Switzerland (SEROCoV-POP): a population-based study. Lancet 2020;396:313–19. 10.1016/S0140-6736(20)31304-0 PMC7289564 32534626

[21] Galindo J. Faltan pruebas para medir el virus (y muchos casos por contar) en Latinoamérica. EL PAÍS 2020. https://elpais.com/sociedad/2020-04-20/faltan-pruebas-para-medir-el-virus-y-muchos-casos-por-contar-en-latinoamerica.html. Accessed 1 Sept 2020.

[22] Australian Government (2020). Modelling the current impact of COVID-19 in Australia.

[23] Russell TW, Hellewell J, Jarvis CI, van Zandvoort K, Abbott S, Ratnayake R, et al. Estimating the infection and case fatality ratio for coronavirus disease (COVID-19) using age-adjusted data from the outbreak on the Diamond Princess cruise ship, February 2020. Euro Surveill. 2020;25(12). 10.2807/1560-7917.ES.2020.25.12.2000256. 10.2807/1560-7917.ES.2020.25.12.2000256 PMC7118348 32234121

[24] Shim E, Mizumoto K, Choi W, Chowell G (2020). Estimating the risk of COVID-19 death during the course of the outbreak in Korea, February–May 2020. J Clin Med.

[25] Mizumoto K, Kagaya K, Zarebski A, Chowell G (2020). Estimating the asymptomatic proportion of coronavirus disease 2019 (COVID-19) cases on board the Diamond Princess cruise ship, Yokohama, Japan, 2020. Eurosurveillance..

[26] Emery JC, Russell TW, Liu Y, Hellewell J, Pearson CA, CMMID COVID-19 Working Group, et al. The contribution of asymptomatic SARS-CoV-2 infections to transmission on the Diamond Princess cruise ship. eLife. 2020;9:e58699. 10.7554/eLife.58699 PMC7527238 32831176

[27] Anonymous. Coronavirus: Iran cover-up of deaths revealed by data leak. BBC News. 2020. https://www.bbc.co.uk/news/world-middle-east-53598965. Accessed 1 Sept 2020.

[28] Preliminary analysis of SARS-CoV-2 importation & establishment of UK transmission lineages. Virological. 2020. https://virological.org/t/preliminary-analysis-of-sars-cov-2-importation-establishment-of-uk-transmission-lineages/507. Accessed 1 Sept 2020.

