# Supplementary material for: Reconstructing the early global dynamics of under-ascertained COVID-19 cases and infections
Source: BMC Med. 2020 Oct 22;18:332. doi: 10.1186/s12916-020-01790-9 (PMC7577796; doi:10.1186/s12916-020-01790-9)
Supplement: Supplementary file 1 — Additional file 1 : Supplementary Appendix 1–4. Figure S1: Figure S1. Temporal variation in under-reporting for all countries with greater than 10 deaths for more than 50 days. Figure S2: Temporal variation in testing effort for all countries there was data for in the Our World In Data database [18]. Figure S3. the relationship between case ascertainment and testing effort. We define testing effort as the 7-day moving average of the number of new tests per new case each day. We plot the under-ascertainment estimates along with the testing effort estimates for all countries we have both data for. We then fit, using a loess curve to highlight the positive but weak relationship (, where is Kendall’s rank coefficient). Figure S4. Temporal variation in under-ascertainment and testing effort for the nine countries with the maximum total cases that we have reliable testing effort estimates for. This figure differs from Fig. 1 as the results are computed using the indirectly age-adjusted baseline CFR for each country. Figure S5. Confirmed case curves adjusted for temporal under-ascertainment adjusted indirectly for age. The results are similar to those in Fig. 2 but have been computed using an indirectly age-adjusted baseline CFR for each country. Figure S6. Estimated infection prevalence curves compared with observed seroprevalence data. The results are similar to those in Fig. 3 but have been computed using an indirectly age-adjusted baseline CFR for each country. Figure S7. Temporal variation in under-reporting for all countries with greater than 10 deaths for more than 50 days. The results are similar to those in Figure S1 but have been computed using an indirectly age-adjusted baseline CFR for each country. Table S1. A summary of the country-level serological studies we used for comparison against our model estimates. Table S2. A summary of the city-level or regional-level serological studies we used for comparison against our model estimates. Table S3. A summary of th [file 12916_2020_1790_MOESM1_ESM.pdf]

Supplementary Appendix for
Reconstructing the early global dynamics of unreported COVID-19 cases and infections

Timothy W. Russell[1], Nick Golding[2], Joel Hellewell[1], Sam Abbott[1], Kevin van Zandvoort[1], Christopher I Jarvis[1], Hamish Gibbs[1], Yang Liu[1], Rosalind M. Eggo[1], W. John Edmunds[1], Adam J. Kucharski[1], CMMID COVID-19 working group*

[1]Centre for Mathematical Modelling of Infectious Diseases, London School of Hygiene & Tropical Medicine
[2]Telethon Kids Institute and Curtin University, Perth, Western Australia

** Corresponding author: timothy.russell@lshtm.ac.uk*

CMMID COVID-19 working group members (order selected at random):

1. Estimating under-ascertainment rates

Daily under-ascertainment calculation

To calculate the level of under-ascertainment on a given day t in country c, first we estimate a delay-adjusted number of cases with outcomes known by time t; $\mathrm{dC}_{c,t}$. This delay-adjustment uses a discrete convolution correction method, accounting for all cases which to-date do not have known outcomes. Specifically, the correction term, $\mathrm{dC}_{c,t}$ for the proportion of cases with known outcomes on day t is given by

$$\mathrm{dC}_{c,t} = \sum_{j=0}^{t} \mathrm{C}_{c,t-j}\, g_j ,$$

where $\mathrm{C}_{c,t}$, the daily national case incidence and g_t the proportion of cases with known outcomes at time t after confirmation. Specifically, g_t represents the probability density function between confirmation-to-death, discretised between time-points using whichever time-resolution the data is on - typically days. We use a hospitalisation-to-death distribution approximated by a lognormal distribution with a mean of 13 days (8.7 - 20.9 days) and standard deviation of 12.7 days (6.4 - 26 days) (1) (see Table S3 for more details on this distribution and the other model parameters).

Let $a_{c,t}$ denote the level of ascertainment at each time in each country. An estimator for the proportion of symptomatic cases ascertained on a given day is:

$$\hat{a}_{c,t} = \frac{\mathrm{bCFR}}{\mathrm{dCFR}_{c,t}},$$

where bCFR is the baseline case fatality ratio and $\mathrm{dCFR}_{c,t} = \mathrm{D}_{c,t}/\mathrm{dC}_{c,t}$ is the delay-adjusted case fatality ratio in that time and country, given by the ratio of daily deaths $\mathrm{D}_{c,t}$ to cases for which the outcome (death or survival) would be known by that time. However this point-wise estimator does not enable robust estimation of time-varying ascertainment rates.

2. Fitting temporal trend with a Gaussian process

ascertained cases and the apparent ascertainment rate $a^*_{c,t}$. Ie. the correction to the number of ascertained cases is applied in the model likelihood. We define the time-varying apparent ascertainment rates as:

$$\Phi^{-1}(a^*_{c,t}) = f_c(t) + \epsilon_{c,t},$$
$$\epsilon_{c,t} \sim N(0, \sigma^2_{c,1}),$$

where $f_c(t)$ is a nonparametric function of time for country c, $\epsilon_{c,t}$ are independent daily noise terms, and $\Phi^{-1}(x)$ is the inverse of the probit function, which maps function values to the unit interval - the range of supported values of the ascertainment rate. We model $f_c(t)$ as a realisation of a univariate zero-mean Gaussian process:

$$f_c(t) \sim \mathcal{GP}(\mathbf{0}, k(t, t'; \theta_c)),$$

with additive covariance function $k(t, t'; \theta_c)$ given by the sum of two component covariance functions (implying summation of their resulting covariance matrices): k_{bias}, a 'bias kernel' modelling the average value of $a_{c,t}$ over the whole period, and a squared exponential covariance function modelling temporal variation in ascertainment about that mean. These covariance functions are defined as:

$$k_{\text{bias}}(t, t') = \sigma^2_{c,2} \ ,$$
$$k_{SE}(t, t') = \sigma^2_{c,3} e^{-\frac{1}{2l_c^2}\|t-t'\|^2} \ .$$

Note this summation of covariance functions is equivalent to defining $f_c(t)$ as the sum of a single squared exponential covariance function and an intercept term with zero-mean normal prior with variance $\sigma^2_{c,2}$. Whilst this compositional Gaussian process representation is uncommon outside the Gaussian process machine-learning literature, it is computationally more convenient since it marginalises out an intercept parameter that would otherwise be poorly identified and lead to a correlated posterior density that would be difficult to sample from.

We consider that the Gaussian process represents the 'signal' in the apparent ascertainment rate; the true ascertainment rate $a_{c,t}$, and that the independent Gaussian error reflects noise in the apparent ascertainment rate over time, capturing extra-Poisson stochasticity (akin to a Poisson-lognormal model of overdispersion) in the time series of reported deaths, such as clustering of reporting of deaths. We therefore estimate the time varying ascertainment as:

$$\Phi^{-1}(a_{c,t}) = f_c(t)$$

We define the following prior distributions over the kernel and error parameters for each country:

$$\sigma_{c,1} \sim N^+(0, 0.5^2)$$

$$\log(\sigma_{c,3}) \sim N(1, 1^2)$$
$$\log(l_c) \sim N(4, 0.5^2)$$

where $N^{+}(.,.)$ denotes a positive-truncated normal distribution, and we set the prior variance for the bias kernel (intercept term) to $\sigma_{c,2} = 1$.

3. Choice of priors

The prior variance of 1 on the bias kernel (intercept term), $k_{\text{bias}}(t, t') = \sigma^2_{c,2}$, corresponds to a uniform prior on 0-1 for ascertainment holding the other components at zero (the probit link is the CDF of the standard normal, so a probit transformation of a standard normal yields a standard uniform distribution).

The half normal prior on the error variance, $\epsilon_{c,t} \sim N(0, \sigma^2_{c,1})$, is a standard shrinkage prior that constrains the residual IID errors to 0, or small values in the absence of evidence to the contrary.

The lognormal prior on the GP amplitude parameter, $\sigma^2_{c,3}$, does not shrink towards zero, since that would imply a prior assumption that there is no temporal correlation. Similarly, we use a lognormal prior on the lengthscale parameter, l_c, since a prior mass close to zero implies very rapid changes in the ascertainment rate. The two lognormal priors were chosen manually to enable a wide range of 'shapes' of the ascertainment rate, without leading to long periods of ascertainment at the boundaries (close to 1 or zero, since the probit links squishes large positive/negative values toward those). I.e. they were chosen to be minimally informative.

Finally, we incorporate uncertainty around the assumed baseline CFR by treating it as a random variable with an informative prior. Specifically, we assume it is normally distributed with mean and SD matching the reported CIs (2), and truncated from 0% to 100%.

2. Numerical procedure

We fit the model by Hamiltonian Monte Carlo using the R packages greta and greta.gp (3). Each model was fitted with 500 independent MCMC chains (a computationally-efficient strategy to yielding large numbers of posterior samples) of 10000 samples each, after discarding an initial 1000 samples per chain during a warm up period during which the sampler was tuned. Using these 500,000 posterior samples, we estimated the posterior median of the posterior and 95% credible interval (CrI) for each time point (black filled line for median and blue shaded region for 95% CrI in Figure 1 and Figure S1).

We assessed convergence of the chains using the Gelman–Rubin convergence diagnostic. Specifically, we tested whether $\hat{R} \leq 1.1$ and whether $\min(n_{\text{eff}}) > 1000$ across all chains. Once these conditions were satisfied, we assumed convergence to the posterior.

3. Data

The input data for the model is a time-series of new cases and new deaths. The temporal and spatial resolution of the input data directly reflects the resolution of the resulting estimates. I.e. if the input data corresponds to the new cases and new deaths each day for a country, then we are able to estimate the under-ascertainment each day for that country. The spatial resolution is important for the accuracy of the estimates, given that some countries have highly heterogeneous population distributions, with concentrated outbreaks in large cities. We therefore use regional data, where it is available for direct comparisons with seroprevalence data (Figure 3) in such countries. We typically find that the accuracy of the estimates increases as the spatial resolution of the input data increases. Unfortunately regional data is not as easy to find from a centralised and regularly updated source.

For the new cases and new deaths time-series data required as a model input, we use the publicly available data from the European Centre for Disease Control (found here: https://www.ecdc.europa.eu/en/publications-data/download-todays-data-geographic-distribution-covid-19-cases-worldwide), which is updated daily. Countries must have had at least ten deaths, for longer than ten days, for their estimates to be computed. Fewer deaths, or for a few days (or both) results in spurious estimates with 95% credible intervals that typically range from 0-100% of the cases ascertained.

4. Further details on methodology and limitations

Baseline CFR

We assumed the age-adjusted baseline CFR is 1.4% (95% CrI: 1.2% - 1.5%) (4), which is broadly consistent with other published estimates (2,4,5) and assumed a range of 10% - 70% of infections were asymptomatic (6–9) with a mean value of 50% (10). Given the uncertainty in these estimates, we propagated the variance in baseline CFR and range in proportion asymptomatic in the inference process so the final 95% credible interval reported for under-ascertainment reflects underlying uncertainty in the model parameters. We also assumed that deaths from COVID-19 are accurately reported. If local testing capacity is limited, or if testing policy affects attribution of deaths (for example, the evidence for the efficacy of post-mortem swabbing is lacking), deaths can be misattributed to a cause other than COVID-19. In that case, our model may underestimate the true burden of infection. However, our estimates were consistent with published serological data. Given that our estimates of under-ascertainment in many countries suggest that the numbers of symptomatic infections at the peak of the outbreak were an order of magnitude larger than reported cases, even if deaths are under-reported, our estimates are still likely to be much closer to the true burden than locally reported cases imply. Our estimates of under-ascertainment over time require a time-series of COVID-19 deaths as an input, a data source that may also exhibit reporting variation. One significant example of this was Spain during June 2020 (Figure S1). However, as our Gaussian process model quantifies time-varying case ascertainment, it is able to account for positive or negative spikes in reporting (11). Specifically, we are able to infer what are known as the inducing points of the temporal trends: the most likely times trends in the under-ascertainment estimates change qualitatively (see the Extended Methods section in the Supplementary Material for more details on the model fitting procedure).

Assuming a fixed baseline CFR of 1.4% (95% CrI: 1.2% - 1.5%) means that we are not accounting for the differences in underlying age distributions between different countries. It is well-known that the

severity of COVID-19 has a strong age-dependency (2). Therefore, it is likely that in countries with younger-skewed populations that we overestimate the ascertainment rate in such countries and vice versa in older-skewed countries. We have implemented an indirect age-adjusted baseline CFR for each country, but this comes with its own set of limitations. The main one being that the age-adjusted CFR results were able to be estimated as they assumed a flat attack rate across age-groups (2). In the absence of case and death time series input data stratified by age, we opted for the parsimonious method of a flat baseline CFR across all countries. To investigate the sensitivity of our methods to this flat CFR, we reproduced Figure 3 with the age-adjusted ascertainment estimates (Figure S7).

Proportion of infections that are asymptomatic

Adjusting for the true number of asymptomatic was performed by simply assuming a wide range, reflecting the still-present uncertainty in the literature of 10-70% of all infections. This proportion scales the adjusted case curves. The proportion of asymptomatic/pre-symptomatic infections has been estimated to also vary with age (12). Again, in the absence of age-stratified data globally, we opt for a simple adjustment, which is equivalent across all settings. As more detailed data comes in, it would be possible to refine and improve the accuracy of the methods presented.

Reporting caveats including under-reporting of deaths

We use data on reported deaths, but these values may represent different events across different countries. For example, some countries did not initially report deaths from care homes (13). There have also been instances of data being retrospectively updated, such as when Spain recorded a negative value of -1918 deaths on the 26th May. Our methods account for this temporal variation by considering the using the Gaussian process to represent the 'signal' in the apparent ascertainment rate, capturing extra-Poisson stochasticity (akin to a Poisson-lognormal model of overdispersion) in the time series of reported deaths, such as clustering of reporting of deaths (See subsection Fitting temporal trend with a Gaussian process for more details). However, the large spike in the Spanish data was outside the range of routine modelled day-to-day variation and so the resulting CrI of our estimates were inconsistent with observed dynamics. We therefore only ran inference on data up until the 26th May and did not include later dates. A more detailed analysis on the Spanish dataset could redistribute the large negative number of deaths to surrounding days, such that the model could deal with the negative deaths more accurately.

Time delay assumptions

There are multiple time delays during the reporting process, from confirmation to hospitalisation to death (14). When estimating cumulative incidence within a country and presenting it as a percentage of the total population, we adjust the reported case curves for under-ascertainment and potential asymptomatic infections. In doing so, we are attempting to describe the number infected at point of infection rather than point of confirmation. To do so, we mean-shift the dates by the mean of the distribution between onset of symptoms and confirmation (15). The distribution has a mean of 9 days. Mean-shifting is a crude adjustment, with known errors. In doing so, we assume that reporting delays are static over-time and equivalent for all countries. Given that we are performing analysis globally, other more complex methods were not opted for as they would have incurred substantial computational costs on top of the computationally intensive Gaussian process framework. Further, between-country variation in delays until confirmation would need to be considered if a more detailed approach were taken, which would require more detailed data than is known to exist for most

countries. However, as we only report infection time as cumulative incidence, a substantial portion of the individual variation in infection time would not be reflected in the incidence, as only the dates which truly occurred before the mean of the delay distribution would be incorrect (those occurring after the mean of the distribution would already be included in the cumulative count). More accurate estimation may be possible with good progress made attempting to solve imputing infection dates from date-of-confirmation data (16). Such methods were motivated by Gostic et al. (14) and are able to accurately reconstruct the true infection curves, validated against simulated data.

Supplementary Figures

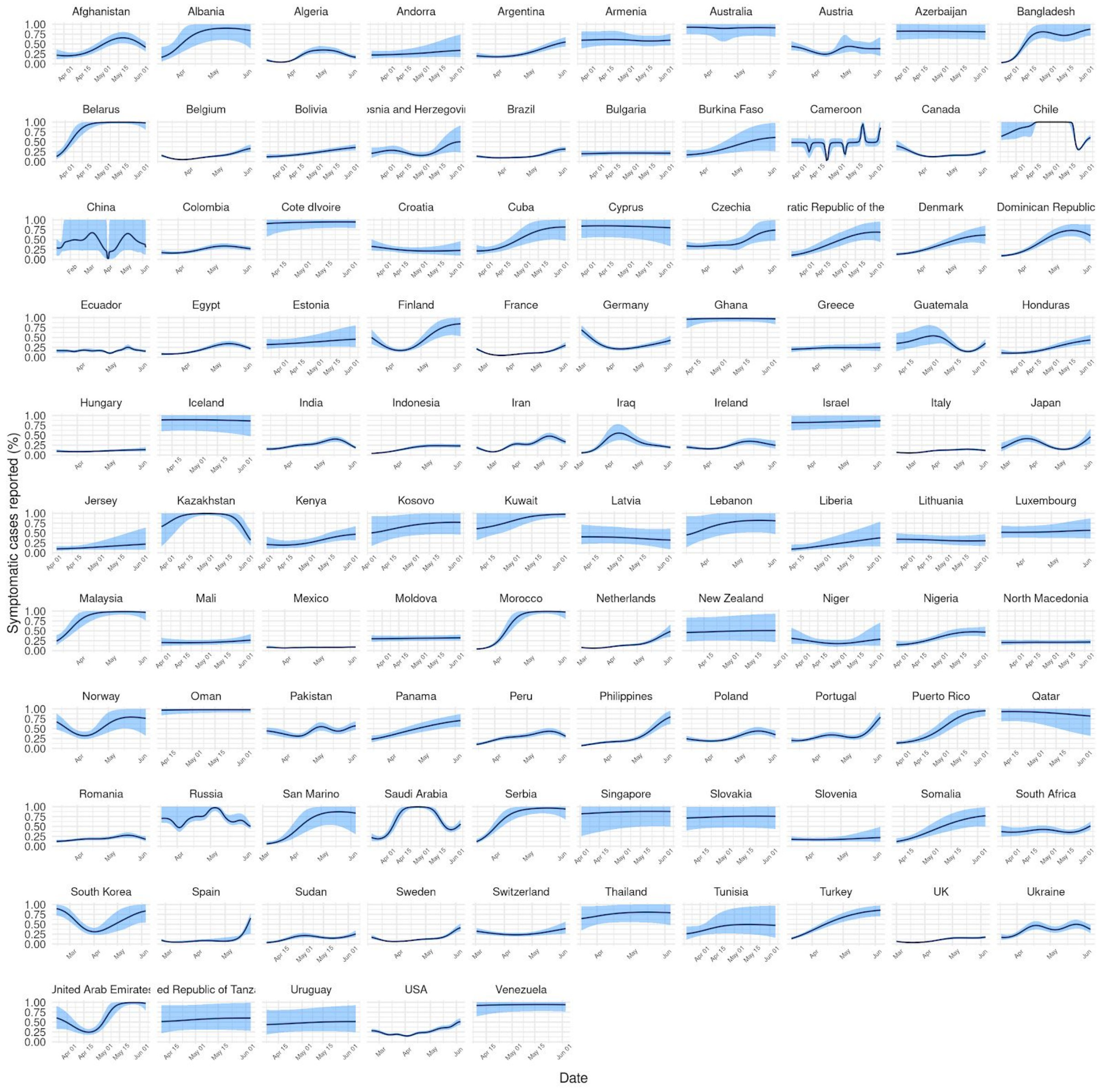

Figure S1: Temporal variation in under-reporting for all countries with greater than 10 deaths for more than 50 days.

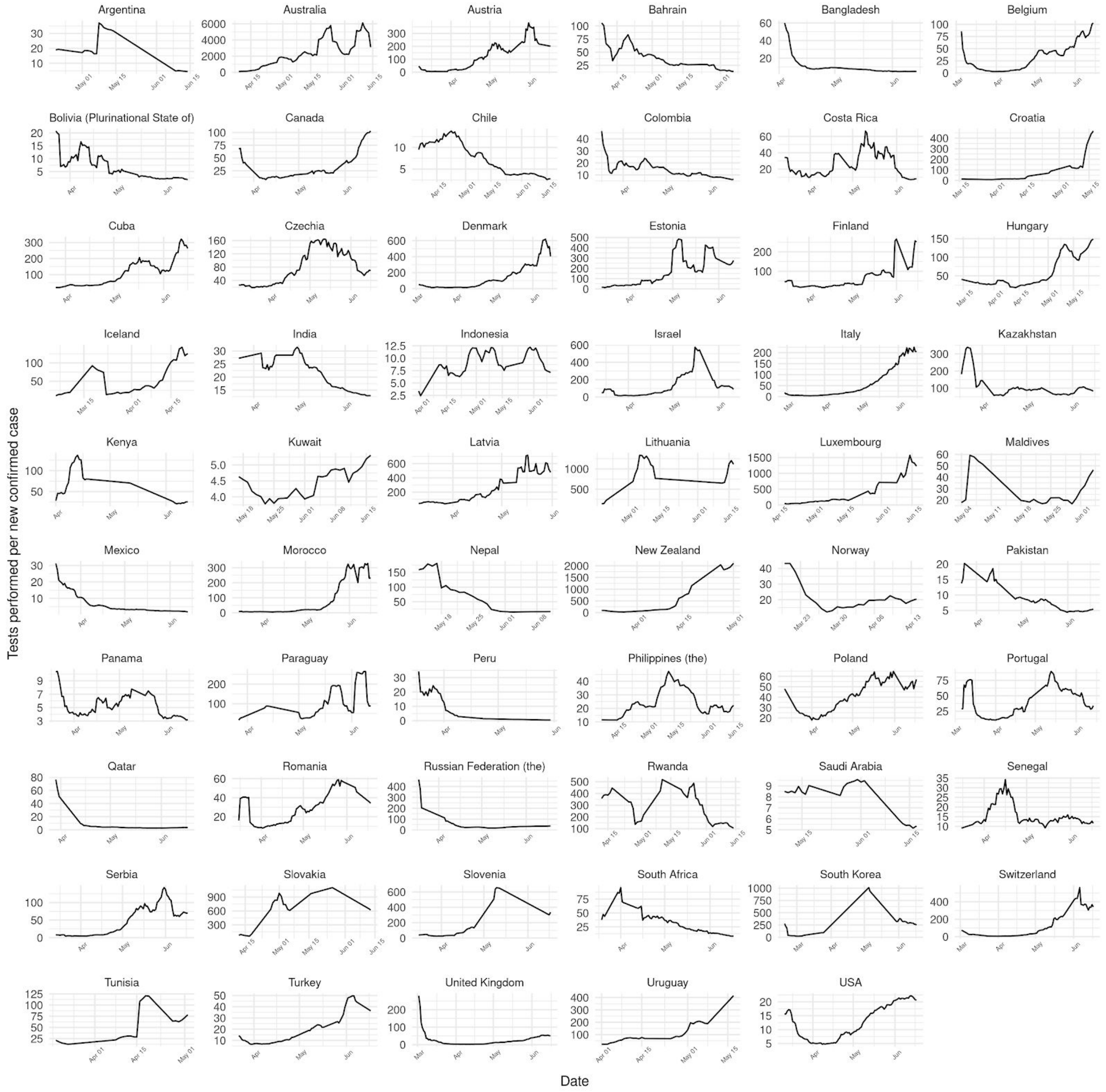

Figure S2: Temporal variation in testing effort for all countries there was data for in the Our World In Data database (17).

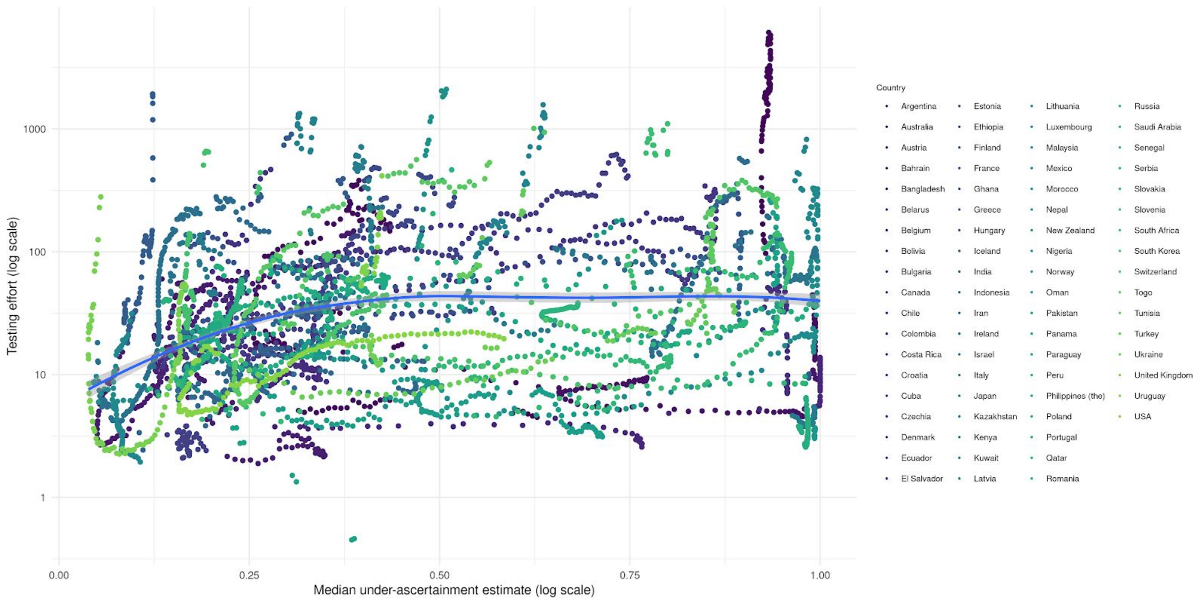

Figure S3: the relationship between case ascertainment and testing effort. **We define testing effort as the 7-day moving average of the number of new tests per new case each day. We plot the under-ascertainment estimates along with the testing effort estimates for all countries we have both data for. We then fit, using a loess curve to highlight the positive but weak relationship ($\tau = 0.16$, where τ is Kendall's rank coefficient).**

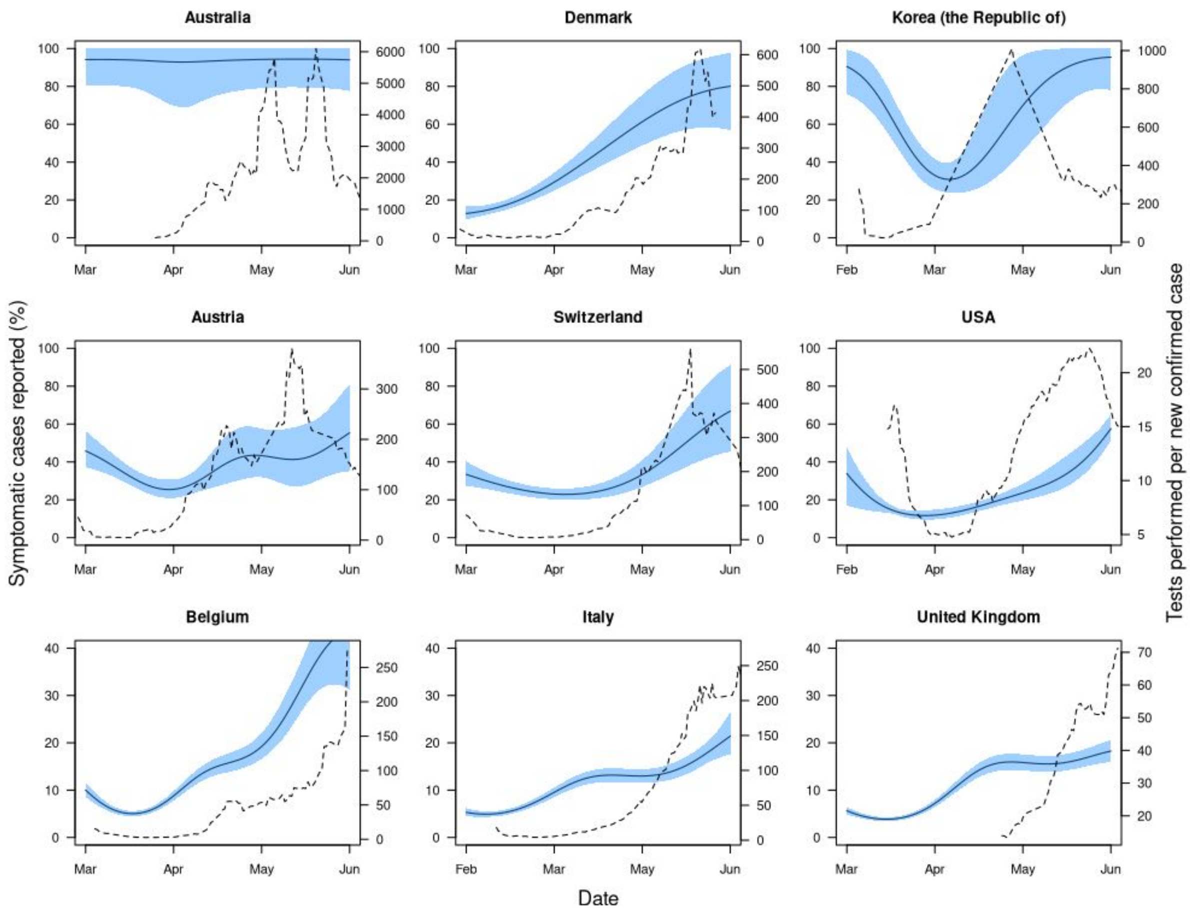

Figure S4: Temporal variation in under-ascertainment and testing effort for the nine countries with the maximum total cases that we have reliable testing effort estimates for. **This figure differs from Figure 1 as the results are computed using the indirectly age-adjusted baseline CFR for each country.**

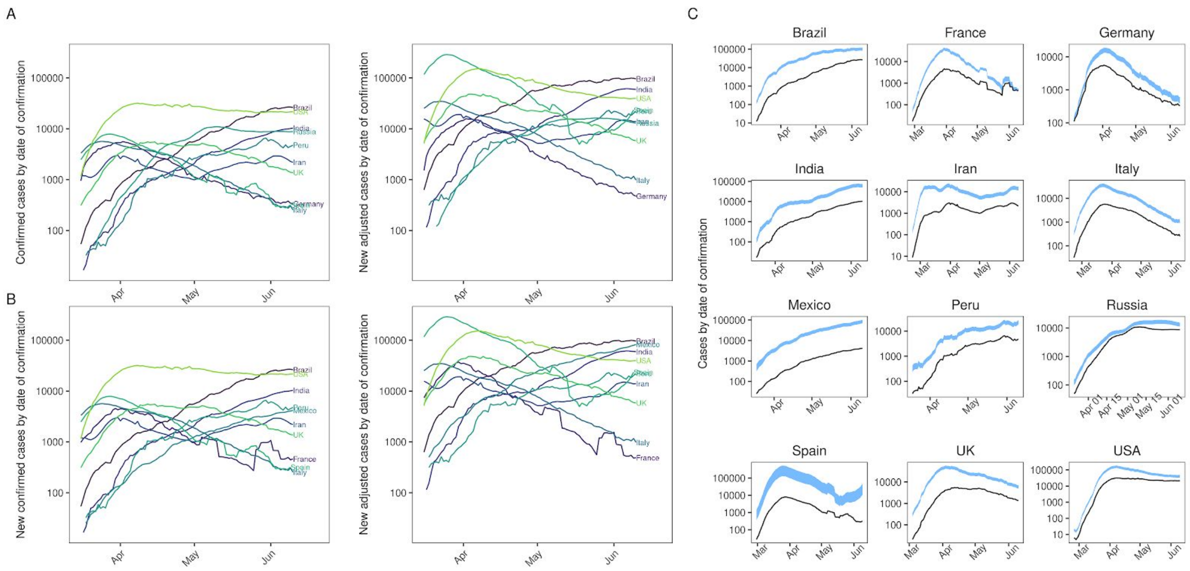

Figure S5: Confirmed case curves adjusted for temporal under-ascertainment adjusted indirectly for age. **The results are similar to those in Figure 2 but have been computed using an indirectly age-adjusted baseline CFR for each country.**

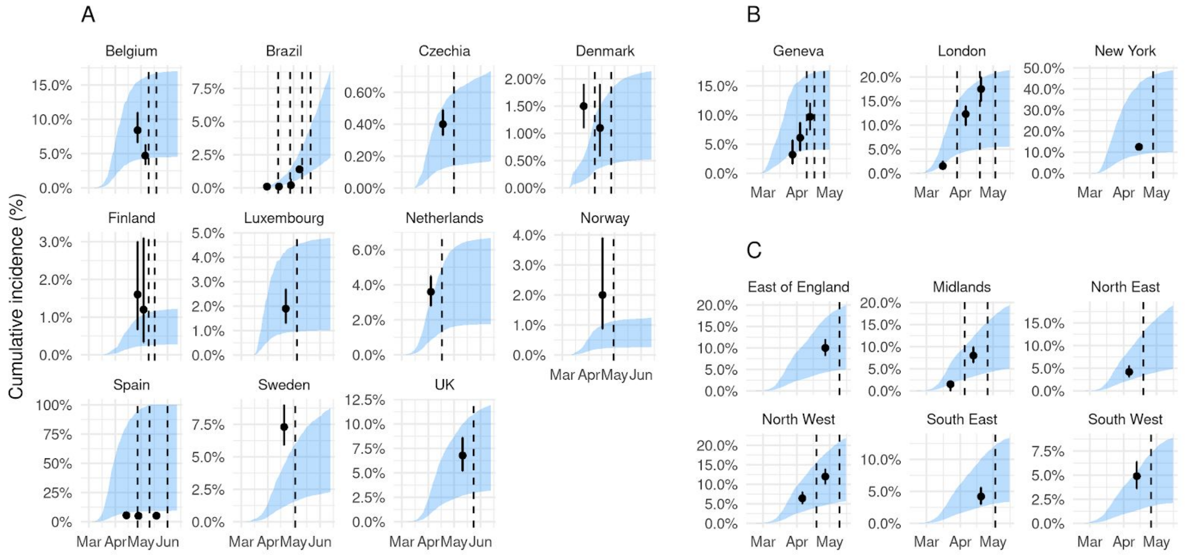

Figure S6: Estimated infection prevalence curves compared with observed seroprevalence data. **The results are similar to those in Figure 3 but have been computed using an indirectly age-adjusted baseline CFR for each country.**

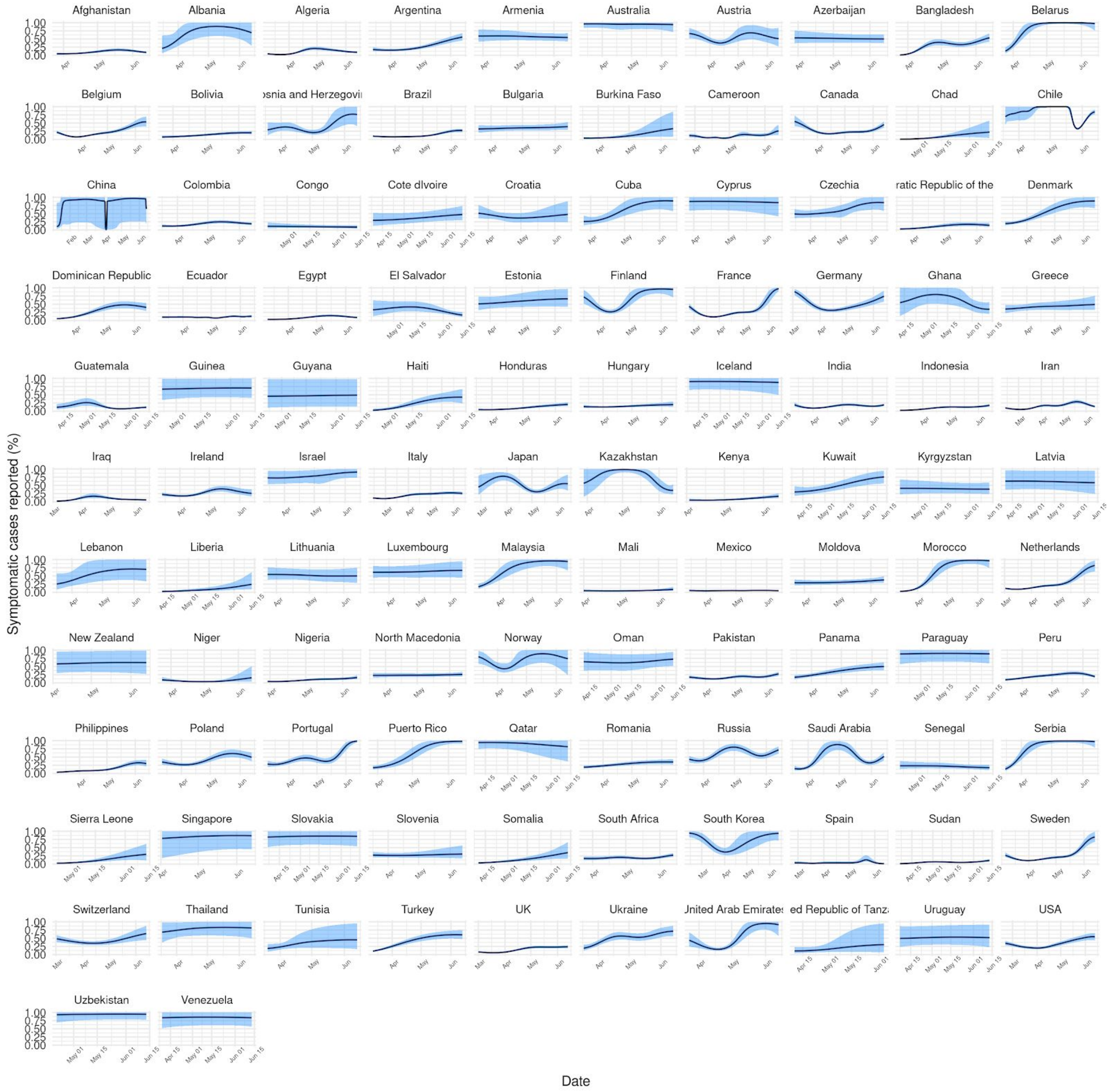

Figure S7: : Temporal variation in under-reporting for all countries with greater than 10 deaths for more than 50 days. **The results are similar to those in Figure S1 but have been computed using an indirectly age-adjusted baseline CFR for each country.**

Country	Sampling end date	Percentage positive (95% CI)	Source
Andorra	13th May	8.5% (8.3% - 8.7%)	Martinez Benazet agraeix a institucions, voluntaris ia la població que ha participat en l'estudi nacional d'anticossos
Belgium	10th May	8.4% (6.6% - 11%)	COVID-19 – WEKELIJKS EPIDEMIOLOGISCH BULLETIN VAN 29 MEI 2020 INHOUDSTAFEL
Belgium	19th May	4.7% (3.4% - 6.3%)	COVID-19 – WEKELIJKS EPIDEMIOLOGISCH BULLETIN VAN 29 MEI 2020 INHOUDSTAFEL
Brazil	13th Apr	0.1% (0.01% - 0.17%)	Apresentação do PowerPoint
Brazil	27th Apr	0.1% (0.05% - 0.29%)	Apresentação do PowerPoint
Brazil	11th May	0.2% (0.27% - 0.69%)	Apresentação do PowerPoint
Brazil	21st May	1.4% (1.2% - 1.5%)	Remarkable variability in SARS-CoV-2 antibodies across Brazilian regions: nationwide serological household survey in 27 states
Czech Republic	1st May	0.4% (0.33$ - 0.49%)	https://koronavirus.mzcr.cz/infekce-covid-19-prosla-ceskou-populaci-velmi-mirne-podobne-jako-v-okolnich-zemich/
Denmark	8th Apr	1.5% (1.1% - 1.9%)	Estimation of SARS-CoV-2 infection fatality rate by real-time antibody screening of blood donors
Denmark	27th Apr	1.1% (0.58% - 1.9%)	Notat: Foreløbige resultater fra den repræsentative seroprævalensundersøgelse af COVID-19. Den 20. maj 2020
Finland	10th May	1.6% (0.67% - 3.0%)	Koronaepidemian väestöserologiatutkimuksen viikkoraportti
Finland	17th May	1.2% (0.33% - 3.1%)	Koronaepidemian väestöserologiatutkimuksen viikkoraportti
Luxembourg	5th May	1.9% (1.3% - 2.7%)	Prevalence of SARS-CoV-2 infection in the Luxembourgish population: the CON-VINCE study.
Netherlands	17th Apr	3.6% (2.8% - 4.5%)	Children and COVID-19
Norway	30th Apr	2% 0.87% - 3.9%)	Truleg berre ein liten andel som har vore smitta av koronavirus i Noreg
Spain	27 Apr	5.5% (3.2% - 8.6%)	Primer estudi que revela la protecció de la nostra població davant del coronavirus
Spain	11 May	5% (4.8% - 5.2%)	Consumo y Bienestar Social - Gabinete de Prensa - Notas de Prensa
Spain	1 Jun	5.2% (5.0% - 5.4%)	ESTUDIO ENE-COVID19: SEGUNDA RONDA
Sweden	3 May	7.3% (5.9% - 9.0%)	Första resultaten från pågående undersökning av antikroppar för covid-19-virus
UK	24 May	6.78% (5.2% - 8.6%)	Coronavirus (COVID-19) Infection Survey pilot

Table S1: A summary of the country-level serological studies we used for comparison against our model estimates.

Country	City or region	Sampling end date	Percentage positive (95% CI)	Source
UK	East of England	2020-05-10	10% (8.2% - 12%)	Sero-surveillance of COVID-19 - GOV.UK
UK	London	2020-03-29	1.5% (0.84% - 2.5%)	Sero-surveillance of COVID-19 - GOV.UK
UK	London	2020-04-19	12.3% (10% - 14%)	Sero-surveillance of COVID-19 - GOV.UK
UK	London	2020-05-03	17.5% (15% - 20%)	Sero-surveillance of COVID-19 - GOV.UK
UK	Midlands	2020-04-05	1.5% (0.02% - 0.72%	Sero-surveillance of COVID-19 - GOV.UK
UK	Midlands	2020-04-26	8% (6.4% - 9.9%)	Sero-surveillance of COVID-19 - GOV.UK
UK	North East	2020-04-19	4.2% (3.0% - 5.6%)	Sero-surveillance of COVID-19 - GOV.UK
UK	North West	2020-05-10	12% (10% - 14%)	Sero-surveillance of COVID-19 - GOV.UK
UK	North West	2020-04-19	6.4% (5.0% - 8.1%)	Sero-surveillance of COVID-19 - GOV.UK
UK	South East	2020-05-03	4.2% (3.0% - 5.6%)	Sero-surveillance of COVID-19 - GOV.UK
UK	South West	2020-04-26	4.9% (3.6% - 6.4%)	Sero-surveillance of COVID-19 - GOV.UK
Switzerland	Geneva	2020-04-10	3.2% (1.6% - 5.7%)	Repeated seroprevalence of anti-SARS-CoV-2 IgG antibodies in a population-based sample from Geneva, Switzerland
Switzerland	Geneva	2020-04-17	6.1% (3.9% - 8.7%)	https://www.medrxiv.org/content/10.1101/2020.05.02.20088898v1.full.pdf
Switzerland	Geneva	2020-04-26	9.7% (7.4% - 12%)	Repeated seroprevalence of anti-SARS-CoV-2 IgG antibodies in a population-based sample from Geneva, Switzerland
USA	New York State	2020-04-28	12.5% (12% - 13%)	Cumulative incidence and diagnosis of SARS-CoV-2 infection in New York

Table S2: A summary of the city-level or regional-level serological studies we used for comparison against our model estimates.

Parameter	Description	Value (95% CI or CrI if applicable) or prior specification	Literature source (if applicable)
c_t	Number of new cases on day t	N/A	ECDC website (18)
d_t	Number of new deaths on day t	N/A	ECDC website (18)
$\hat{a}_{c,t}$	The proportion of cases ascertained on day t	N/A	N/A
f_t	Discretised probability density of death on day t	Mean: 13 days (8.7 - 20.9)<br>SD: 12.7 days (6.4 - 21.8)	Linton et al. (2020) (1)
bCFR	The assumed baseline CFR	1.4% (1.2% - 1.5%)	Verity et al. (2020) (2)
$\text{dCFR}_{c,t}$	The country specific delay-adjusted CFR	N/A	N/A
$\sigma^2_{c,2}$	Bias term in kernel of GP	$\sigma^2_{c,2} \sim \text{Uniform}(0,1)$	N/A
$\epsilon_{c,t}$	Error variance term in GP	$\epsilon_{c,t} \sim N(0,\sigma^2_{c,1})$	N/A
$\sigma^2_{c,3}$	GP amplitude parameter	$\log(\sigma_{c,3}) \sim N(1,1^2)$	N/A
l_c	Lengthscale parameter	$\log(l_c) \sim N(4,0.5^2)$	N/A

Table S3: A summary of the parameters, distributions and output quantities either as inputs or outputs of our under-ascertainment model

References

1. Linton NM, Kobayashi T, Yang Y, Hayashi K, Akhmetzhanov AR, Jung S, et al. Incubation Period and Other Epidemiological Characteristics of 2019 Novel Coronavirus Infections with Right Truncation: A Statistical Analysis of Publicly Available Case Data. J Clin Med. 2020 ;9(2):538.
2. Verity R, Okell LC, Dorigatti I, Winskill P, Whittaker C, Imai N, et al. Estimates of the severity of coronavirus disease 2019: a model-based analysis. Lancet Infect Dis. 2020 Jun 1;20(6):669–77.
3. Golding N. greta: simple and scalable statistical modelling in R. J Open Source Softw. 2019 12;4(40):1601.
4. Russell TW, Hellewell J, Jarvis CI, van Zandvoort K, Abbott S, Ratnayake R, et al. Estimating the infection and case fatality ratio for coronavirus disease (COVID-19) using age-adjusted data from the outbreak on the Diamond Princess cruise ship, February 2020. Eurosurveillance. 2020 ;25(12).
5. Shim E, Mizumoto K, Choi W, Chowell G. Estimating the Risk of COVID-19 Death During the Course of the Outbreak in Korea, February–May 2020. J Clin Med. 2020;9(6):1641.
6. Stringhini S, Wisniak A, Piumatti G, Azman AS, Lauer SA, Baysson H, et al. Seroprevalence of anti-SARS-CoV-2 IgG antibodies in Geneva, Switzerland (SEROCoV-POP): a population-based study. The Lancet. 2020 Jun;S0140673620313040.
7. Mizumoto K, Kagaya K, Zarebski A, Chowell G. Estimating the asymptomatic proportion of coronavirus disease 2019 (COVID-19) cases on board the Diamond Princess cruise ship, Yokohama, Japan, 2020. Eurosurveillance. 2020 12;25(10).
8. Emery JC, Russell TW, Liu Y, Hellewell J, Pearson CA, CMMID COVID-19 Working Group, et al. The contribution of asymptomatic SARS-CoV-2 infections to transmission on the Diamond Princess cruise ship. Lipsitch M, editor. eLife. 2020 24;9:e58699.
9. Buitrago-Garcia DC, Egli-Gany D, Counotte MJ, Hossmann S, Imeri H, Ipekci AM, et al. The role of asymptomatic SARS-CoV-2 infections: rapid living systematic review and meta-analysis. Epidemiology; 2020.
10. Rivett L, Sridhar S, Sparkes D, Routledge M, Jones NK, Forrest S, et al. Screening of healthcare workers for SARS-CoV-2 highlights the role of asymptomatic carriage in COVID-19 transmission. van der Meer JW, editor. eLife. 2020 May 11;9:e58728.
11. Tsang TK, Wu P, Yun Lin YL, Lau E, Leung GM, Cowling BJ. Impact of changing case definitions for COVID-19 on the epidemic curve and transmission parameters in mainland China. Epidemiology; 2020.
12. Davies NG, Klepac P, Liu Y, Prem K, Jit M, Eggo RM. Age-dependent effects in the transmission and control of COVID-19 epidemics. Nat Med. 2020 16;1–7.
13. Iacobucci G. Covid-19: Care home deaths in England and Wales double in four weeks. BMJ. 2020 22;369.
14. Gostic KM, McGough L, Baskerville E, Abbott S, Joshi K, Tedijanto C, et al. Practical considerations for measuring the effective reproductive number, Rt. :21.
15. He X, Lau EHY, Wu P, Deng X, Wang J, Hao X, et al. Temporal dynamics in viral shedding and transmissibility of COVID-19. Nat Med. 2020 ;26(5):672–5.
16. Evaluating approaches to backcalculating cases counts by date of infection from cases counts by date of report. Evaluating approaches to backcalculating cases counts by date of infection from cases counts by date of report. 2020. https://github.com/epiforecasts/backcalc/blob/master/report.md
17. Roser M, Ritchie H, Ortiz-Ospina E, Hasell J. Coronavirus Pandemic (COVID-19). OurWorldInData.org. 2020. https://ourworldindata.org/coronavirus
18. Data on the geographic distribution of COVID-19 cases worldwide. European Centre for Disease Prevention and Control. 2020.

https://www.ecdc.europa.eu/en/publications-data/download-todays-data-geographic-distribution-covid-19-cases-worldwide
